# The Differential Formation of the LINC-Mediated Perinuclear Actin Cap in Pluripotent and Somatic Cells

**DOI:** 10.1371/journal.pone.0036689

**Published:** 2012-05-04

**Authors:** Shyam B. Khatau, Sravanti Kusuma, Donny Hanjaya-Putra, Prashant Mali, Linzhao Cheng, Jerry S. H. Lee, Sharon Gerecht, Denis Wirtz

**Affiliations:** 1 Department of Chemical and Biomolecular Engineering, The Johns Hopkins University, Baltimore, Maryland, United States of America; 2 Johns Hopkins Physical Sciences – Oncology Center and Institute for NanoBioTechnology, The Johns Hopkins University, Baltimore, Maryland, United States of America; 3 Department of Biomedical Engineering, The Johns Hopkins University, Baltimore, Maryland, United States of America; 4 Institute for Cell Engineering and Department of Medicine, Johns Hopkins School of Medicine, Baltimore, Maryland, United States of America; 5 Center for Strategic Scientific Initiatives, Office of the Director, National Cancer Institute, National Institutes of Health, Bethesda, Maryland, United States of America; Dalhousie University, Canada

## Abstract

The actin filament cytoskeleton mediates cell motility and adhesion in somatic cells. However, whether the function and organization of the actin network are fundamentally different in pluripotent stem cells is unknown. Here we show that while conventional actin stress fibers at the basal surface of cells are present before and after onset of differentiation of mouse (mESCs) and human embryonic stem cells (hESCs), actin stress fibers of the actin cap, which wrap around the nucleus, are completely absent from undifferentiated mESCs and hESCs and their formation strongly correlates with differentiation. Similarly, the perinuclear actin cap is absent from human induced pluripotent stem cells (hiPSCs), while it is organized in the parental lung fibroblasts from which these hiPSCs are derived and in a wide range of human somatic cells, including lung, embryonic, and foreskin fibroblasts and endothelial cells. During differentiation, the formation of the actin cap follows the expression and proper localization of nuclear lamin A/C and associated linkers of nucleus and cytoskeleton (LINC) complexes at the nuclear envelope, which physically couple the actin cap to the apical surface of the nucleus. The differentiation of hESCs is accompanied by the progressive formation of a perinuclear actin cap while induced pluripotency is accompanied by the specific elimination of the actin cap, and that, through lamin A/C and LINC complexes, this actin cap is involved in progressively shaping the nucleus of hESCs undergoing differentiation. While, the localization of lamin A/C at the nuclear envelope is required for perinuclear actin cap formation, it is not sufficient to control nuclear shape.

## Introduction

The intermediate filament type A lamins, but not type B lamins, are absent from the nuclear lamina in undifferentiated stem cells [1], [2], [3]. Differentiated and undifferentiated cells show strikingly different nuclear shape, plasticity, and mechanical stiffness [2], [4], [5], suggesting that lamin A/C may play a critical role in preventing stem cells from shaping their nucleus. Lamin A/C is connected to the cytoskeleton through linkers of the nucleoskeleton to the cytoskeleton (LINC) complexes, which span the nuclear envelope and mediate physical connections between the nuclear lamina and the cytoskeleton through SUN–KASH interactions [6]. LINC complex SUN domain–containing proteins Sun1 and Sun2 are essential to recruit KASH-domain–containing proteins, including Nesprin 2 giant and Nesprin 3, to the outer nuclear membrane [3], [7], [8], [9], [10], [11], [12].

Recently, it has been shown that mouse embryonic fibroblasts (MEFs) and Swiss 3T3 mouse fibroblasts feature a highly organized actin filament structure that drapes around the nucleus, which we name the perinuclear actin cap [5], [13]. The perinuclear actin cap is composed of thick, contractile, acto-myosin fibers that are tightly connected to the apical surface of the nucleus through components of the LINC complexes [6], [14], [15], [16], [17], [18]. The actin cap, not conventional basal and cortical actin stress fibers, is absent from cells deficient in lamin A/C, a phenotype recapitulated in cells where LINC complexes are specifically displaced from the nuclear envelope to the ER and cytoplasm [5], [13]. Whether undifferentiated stem cells, which lack lamin A/C, feature an actin cap and whether the actin cap contributes to nuclear shaping in stem cells undergoing differentiation are unknown.

Here we show that the perinuclear actin cap is completely absent from both human embryonic stem cells (hESCs) as well as human induced pluripotent stem cells (hiPSCs). In contrast, hESCs undergoing differentiation progressively show an organized actin cap. Similarly, the actin cap is organized in the parental lung fibroblasts from which the iPSCs were derived and in a wide range of human somatic cells. Undifferentiated and differentiated cells all feature conventional basal stress fibers. The formation of the actin cap follows the expression and proper localization of nuclear lamin A/C and associated linkers of nucleus and cytoskeleton (LINC) complex components at the nuclear envelope, which physically couple the highly ordered stress fibers of the actin cap to the apical surface of the nucleus. Furthermore, our results indicate that, through lamin A/C and LINC complexes, the actin cap is involved in properly shaping the nucleus of hESCs undergoing differentiation. These results suggest that the total absence of an actin cap could be a salient feature of pluripotency, that the formation of an actin cap accompanies the differentiation of hESCs, and that the actin cap regulates nuclear shape during hESC differentiation.

## Results

### Differential formation of the perinuclear actin cap in hESCs and human somatic cells

**Figure 1:**
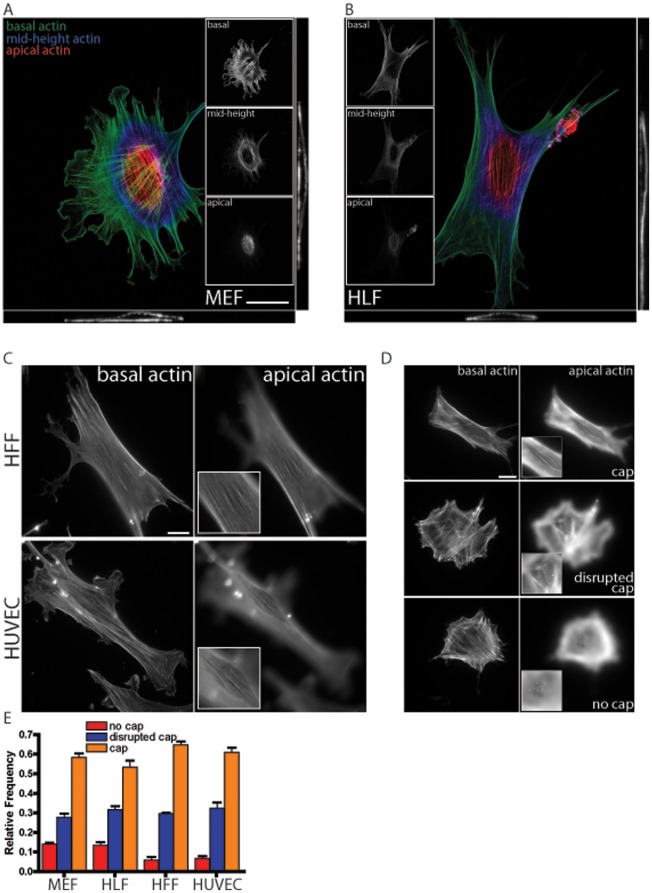
The perinuclear actin cap in human somatic cells. A and B. Confocal microscopy sections (*Insets*) of the actin filament network at the apical surface (red), mid-height (blue), and basal surface (green) of a mouse embryonic fibroblast (MEF, panel A) and a human lung fibroblast (HLF, panel B). The main panel shows the full confocal reconstruction of the three-dimensional actin filament organization. Bottom and side panels show views along the width cross-section (bottom panel) and length cross-section (side panel) through the nucleus. Scale bar, 20 µm. C. Typical organization of the conventional basal stress fibers (left panels) and of the actin cap fibers (right panels) in a human foreskin fibroblast (HFF, top) and a human umbilical vein endothelial cell (HUVEC, bottom), as detected by epifluorescence microscopy. Scale bar, 20 µm. D. Illustrative examples of organized perinuclear actin cap, disorganized actin cap, and no actin cap in a HLF. Scale bar, 20 µm. E. Proportion of MEFs, HLFs, HFFs, and HUVECs showing an organized (orange bars), disrupted (blue bars), and no actin cap (red bars). At least 100 cells in triplicate for a total of 300 cells were probed per condition.

We asked whether human somatic and pluripotent cells differentially formed a perinuclear actin cap. Human lung fibroblasts (HLFs) (the parental cell line used to derive hiPSCs described below) and mouse embryonic fibroblasts (MEFs, which have previously been shown to form a prominent perinuclear actin cap) were stained with phalloidin and DAPI to visualize actin filament organization and nuclear DNA, respectively. Progressively lowering the plane of focus of a confocal microscope from the apical surface of the nucleus down to the underlying substrate and three-dimensional reconstruction of the corresponding actin filament architecture revealed a highly organized ultrastructure wrapping around the interphase nucleus of these two types of cells (Fig. 1, A and B). Similar to HLFs and MEFs, human foreskin fibroblasts (HFFs) and human umbilical endothelial cells (HUVECs) also featured a highly organized perinuclear actin cap (Fig. 1C, right panels). Actin filaments at the apical surface of the nucleus in HFFs (Fig. 1C, top) and HUVECs (Fig. 1C, bottom) formed thick bundles that were mostly parallel to one another in the cap and globally parallel to the direction of the long axis of the nucleus. Actin filament bundles underneath the nucleus were typically less abundant and had no particular orientation with respect to the nucleus or the cell (Fig. 1, A and B, green and top inset; Fig 1C, left panels). In the thin lamella away from the perinuclear region, actin filaments at the basal cellular surface organized in conventional stress fibers that lie completely within the basal region of adherent cells (Fig. 1, A–D). These are the stress fibers that are routinely observed in a wide range of human and rodent somatic adherent cells. MEFs and all three tested types of human somatic cells displayed similar ratios of actin caps to disrupted actin caps to no actin caps (Fig. 1, D and E).

**Figure 2:**
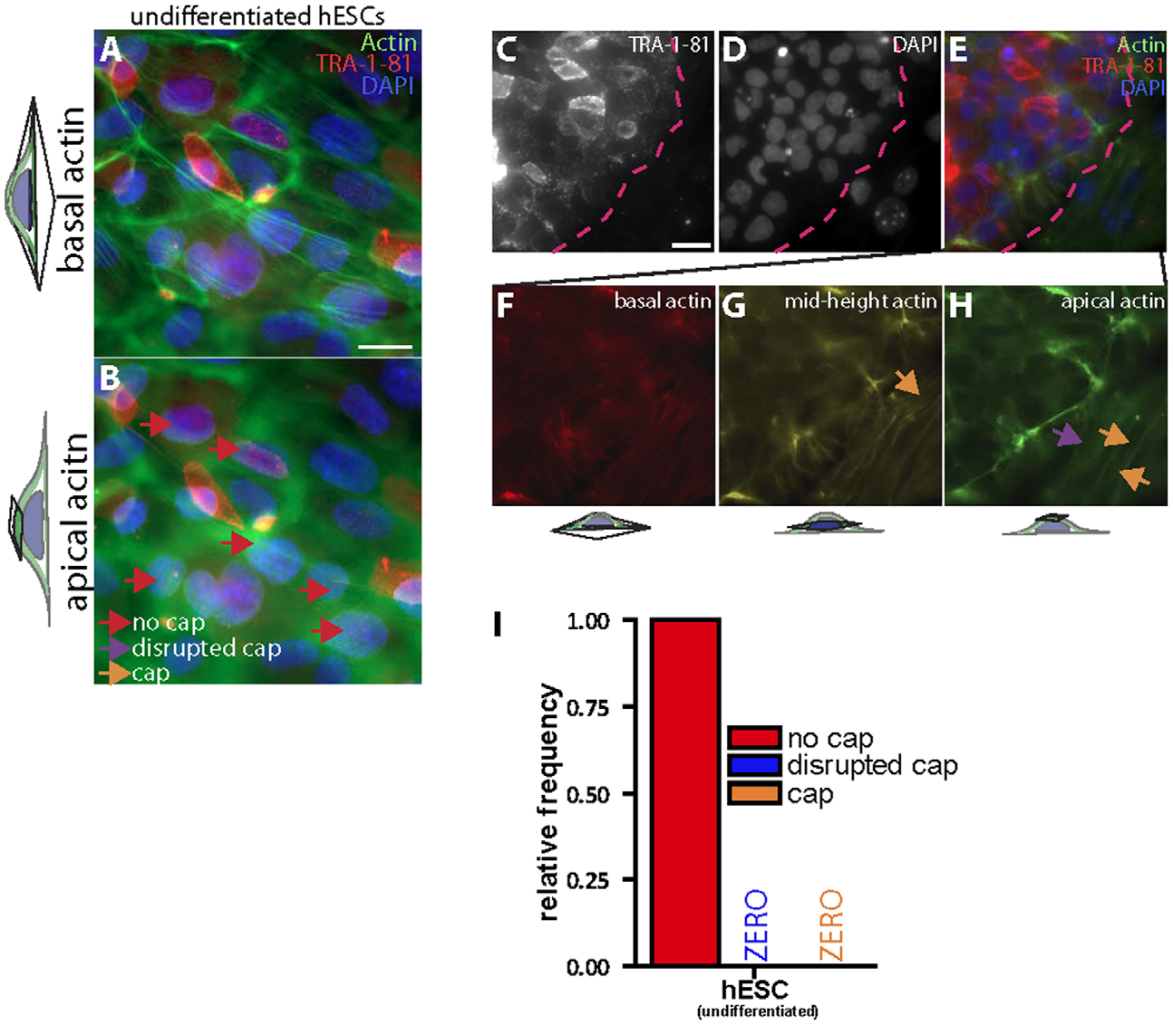
The perinuclear actin cap is absent in undifferentiated human embryonic stem cells (hESCs). A and B. Well developed basal stress fibers (A) and absence of apical perinuclear actin cap (B) in undifferentiated hESCs. Red arrows indicate examples of cells showing no actin cap on top of nuclei. Cells were stained for differentiation marker TRA-1-81 (red), nuclear DNA (DAPI, blue), and actin (green). Scale bar, 20 µm. C–H. Basal and apical actin filament organization in a colony of undifferentiated TRA-1-81-positive hESCs (upper left region of panels C–H) and peripheral TRA-1-81-negative hESCs undergoing differentiation (lower right region of panels C–H). The dashed line delineates the edge of the TRA-1-81-positive hESC colony from TRA-1-81-negative hESCs that have started to undergo differentiation and start forming actin caps. Orange and purple arrows point to organized and disorganized/disrupted actin cap, respectively. Scale bar, 100 µm. I. Fractions of TRA-1-81-positive hESCs showing no actin cap (red bar), a disorganized/disrupted actin cap (blue bar), and an organized actin cap (orange). At least 100 cells in triplicate for a total of 300 cells were probed per condition.

Epifluorescence and confocal fluorescence microscopy were used to examine the organization of actin filaments in undifferentiated H9 human embryonic stem cells (hESCs). hESCs were co-stained with an antibody against tumor rejection antigen 1–81 (TRA-1-81), a standard cell surface marker of pluripotency that is down-regulated during the early phase of differentiation (Fig. S1) [19], [20]. Focus on the subcellular region near the underlying substrate showed prominent, normal stress fibers at the basal surface of undifferentiated hESCs (Fig. 2A). However, remarkably, the apical surface of the nuclei of all examined undifferentiated (TRA-1-81-positive) hESCs were devoid of organized actin filament structure above their nucleus, i.e. undifferentiated hESCs featured no perinuclear actin cap (Fig. 2B). Since cells could be of different thickness and/or at different heights, Z-stack movies were acquired to confirm the lack of actin caps in stem cell colonies as compared to the presence of caps in adjacent remaining feeder MEFs (Fig. 2E). Special attention was paid to use small increments between focal sections (<0.3 µm); the same cells were also scanned starting at slightly different heights as to not miss actin structures underneath the nucleus. Regions of hESC colonies that were TRA-1-81-positive showed no actin caps; regions of the colonies that were TRA-1-81-negative started to form an actin cap (Fig 2, C–H). No undifferentiated, TRA-1-81-positive hESC showed an organized or even disrupted actin cap (Fig. 2I). These results suggest that, contrary to a wide range of human somatic cells, which feature a prominent perinuclear actin cap (Fig. 1), undifferentiated hESCs do not show any perinuclear actin cap structure (Fig. 2).

### The perinuclear actin cap forms progressively in differentiating hESCs

**Figure 3:**
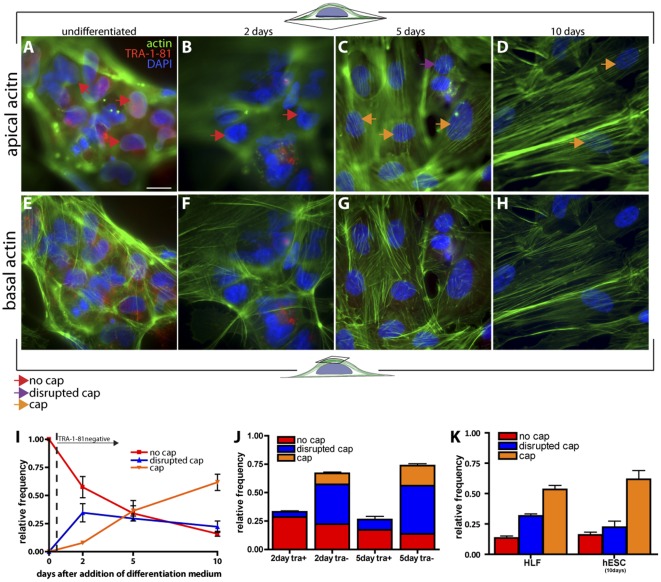
The perinuclear actin cap progressively forms in hESCs following onset of differentiation. A–H. Status of apical perinuclear actin cap (A–D) and basal stress fibers (E–H) in undifferentiated hESCs at day 0 (A and E), as well as two (B and F), five (C and G), and ten days (D and G) after induction of differentiation (+VEGF and collagen IV). Red, purple, and orange arrows indicate examples of cells showing no actin cap, a disrupted/disorganized actin cap, and a well-organized actin cap, respectively. Cells were stained for differentiation marker TRA-1-81, nuclear DNA (DAPI), and actin. Scale bar, 20 µm. I. Evolution of the fraction of TRA-1-81-negative hESCs showing an actin cap. No actin cap was present in TRA-1-81-positive hESCs. J. Proportion of TRA-1-81-positive and TRA-1-81-negative hESCs showing an organized actin cap, a disorganized actin cap, or no actin cap, two and five days after onset of differentiation. K. Distribution of cells showing an actin cap in hESCs after 10 days of differentiation compared to HLFs. At least 100 cells in triplicate for a total of 300 cells were probed per condition.

Following a modification of our previously described differentiation protocol to induce differentiation, hESCs were removed from the feeder cell layer and re-seeded on collagen IV in endothelial growth medium containing vascular endothelial growth factor (VEGF), pushing the cells towards a vascular lineage [21], [22]. For increasing time, a gradually increasing fraction of cells displayed an organized perinuclear actin cap until, after ten days of differentiation, a majority of cells showed a well-organized actin cap (Fig. 3, A–D). Meanwhile, the organization of basal fibers changed due to large cell footprint, but remained qualitatively similar (Fig. 3, E–H). This transition, from complete absence of actin caps in undifferentiated cells (Fig. 2 and Fig. 3A) to the presence of actin caps in a majority of differentiating cells, was progressive (i.e. not abrupt) (Fig. 3, B–D and 3I). We further distilled these results into two groups: TRA-1-81-positive and TRA-1-81-negative cells. In differentiation conditions, no TRA-1-81-positive hESCs showed an organized actin cap (Fig. 3J). An increasing fraction of TRA-1-81-negative cells featured highly parallel stress fibers forming a dome-shaped cap that was gently curved along the vertical axis of the cell and covered the apical surface of the nucleus (Fig. 3, B–D).

After 10 days in differentiation conditions, we observed that more than 84% cells showed an organized perinuclear actin cap (orange arrows, Fig. 3, C and D) or a somewhat disorganized actin cap (purple arrows, Fig. 3C), while 16% showed no actin cap (Fig. 3K). These fractions are nearly identical to the fractions of TRA-1-81-positive (15%) and TRA-1-81-negative cells (85%) 10 days in differentiation conditions. This distribution of actin caps was nearly identical to the actin cap distributions for HLFs (Figs. 1E and 3K) and MEFs (Fig. 1E). These results suggest that, following onset of differentiation, human embryonic stem cells progressively form a perinuclear actin cap until reaching an actin cap distribution nearly identical to actin cap distributions displayed by somatic cells.

We note that the absence of an actin cap was found not to be an intrinsic, long-term property of a subset of somatic cells and differentiated hESCs. Indeed, through live-cell microscopy of GFP-lifeact [13], we found that interphase cells dynamically re-organized their actin cap during motility events, mitotic cells dispensed of their actin cap, and post-mitotic cells took several hours to re-organize their perinuclear actin cap (not shown). This at least partially explained why a non-zero fraction of somatic or differentiated cells in culture showed no actin cap.

### Differential formation of the perinuclear nuclear actin cap in human iPSCs and their parental somatic cells

**Figure 4:**
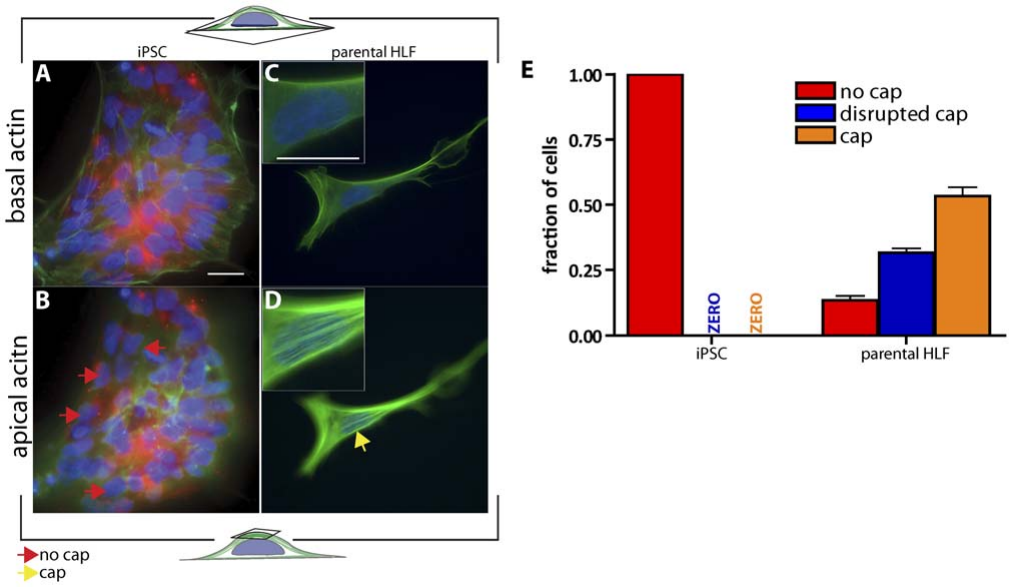
Perinuclear actin cap is absent in human induced pluripotent stem cells (iPSCs), but is present in parental cells from which they are derived. A–D. Typical organization of basal stress fibers (A and C) and perinuclear actin cap (B and D) in iPSCs (A and B) and parental HLFs (C and D) from which they were derived. Red and yellow arrows indicate examples of cells showing no actin cap and a well-organized actin cap, respectively. *Insets*, details of the basal (top inset) and apical (bottom inset) organization of the actin filament network in a HLF. Scale bar, 20 µm. E. Proportion of iPSCs and HLFs showing either an organized actin cap (orange bars), a disrupted actin cap (blue bars), or no actin cap (red bars). At least 200 cells were probed in triplicate for a total of 600 cells for each case.

It has recently been demonstrated that somatic cells can be induced to a pluripotent state through the expression of defined reprogramming genes [23], [24]. We therefore sought to examine whether hiPSCs derived from IMR90 human lung fibroblasts through the expression of *Oct4*, *Sox2*, *NANOG*, and *LIN28* [25] showed the same actin filament architecture as undifferentiated hESCs. Similar to hESCs, hiPSCs in non-differentiating medium showed normal basal actin filament organization, including conventional basal stress fibers (Fig. 4, A and B). Also similar to undifferentiated hESCs, not a single hiPSC featured a perinuclear actin cap (Fig. 4, C and D). In contrast, parental HLFs showed both well-organized basal stress fibers and perinuclear actin caps (Fig. 4, C and D, and insets). The distribution of actin caps in HLFs (Fig. 4E) was again nearly identical to those of hESCs 10 days after onset of differentiation (Fig. 3K) and terminally differentiated MEFs, HFFs, and HUVECs (Fig. 1E), with 15% of HLFs showing no actin cap and 85% showing either an organized actin cap or a somewhat disorganized actin cap. Together these results suggest that the absence of an actin cap is an architectural feature of the actin cytoskeleton shared by both undifferentiated human iPSCs and ESCs and that the progressive appearance of an actin cap strongly correlates with the corresponding differentiated states of these cells.

### LINC complexes are disorganized in undifferentiated hESCs and hiPSCs

**Figure 5:**
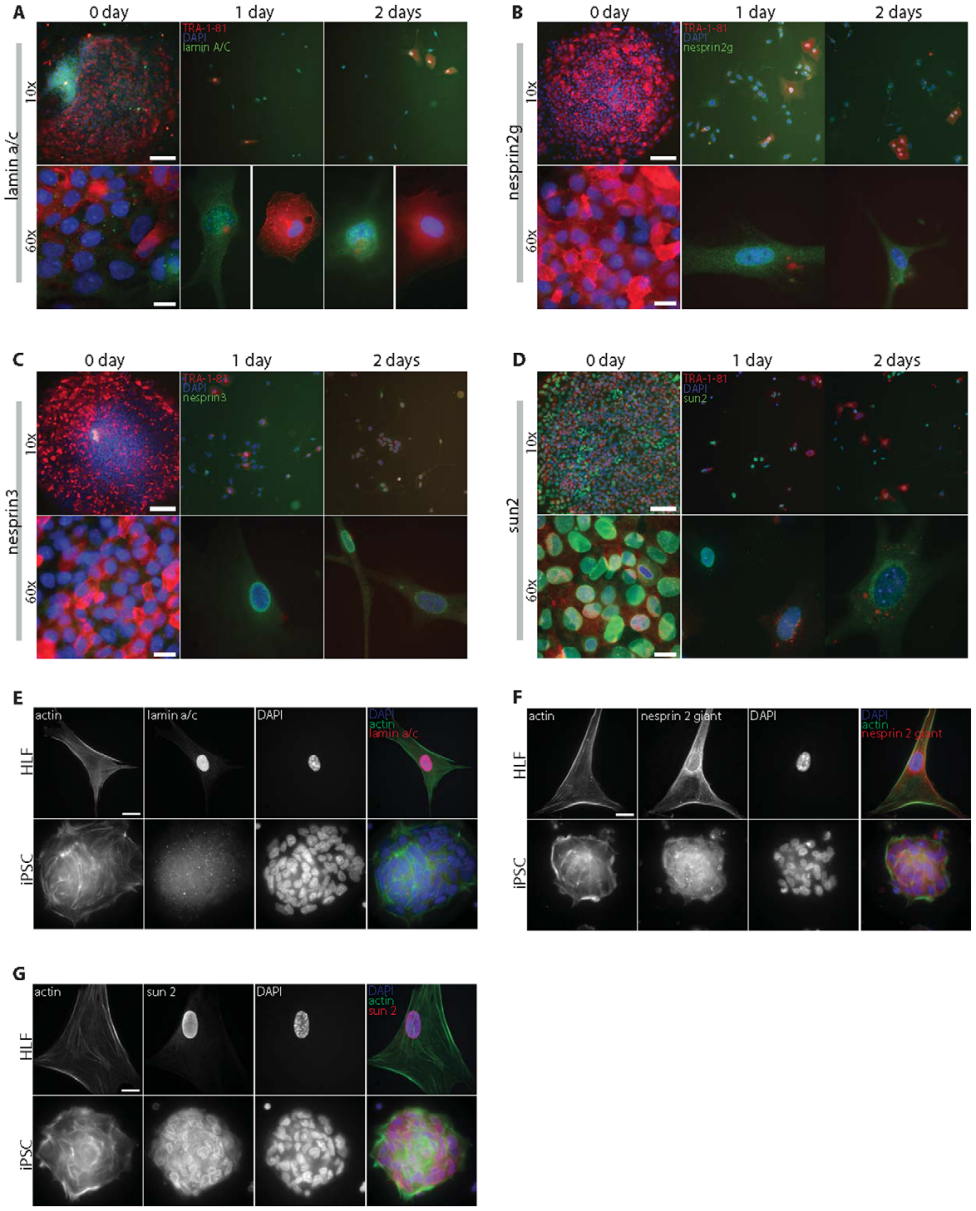
Status of Lamin A/C and LINC complexes in hESCs and iPSC during differentiation. A–D. Low- (10×, top panels) and high-magnification (60×, bottom panels) views of the organization of lamin A/C (A) and LINC complex components Nesprin2 giant (B), Nesprin3 (C), and Sun2 (D) at or near the nuclear envelope in undifferentiated hESCs and hESCs one and two days after switching to differentiation conditions. Cells were stained for nuclear DNA (DAPI), actin, TRA-1-81, and with antibodies against human lamin A/C, Nesprin2 giant, Nesprin3, and Sun2, as indicated, and visualized by immunofluorescence microscopy. Scale bar for 10× micrographs, 100 µm; Scale bar for 60× micrographs, 20 µm. E–G. Immunofluorescence micrographs showing the organization of actin and lamin A/C (E), LINC complex components Nesprin2 giant (F) and Sun2 (G) at the nuclear envelope of parental HLFs (top panels) and IPSCs (bottom panels). Scale bar, 20 µm.

To understand the absence of actin caps in hESCs and iPSCs and the mechanism of formation of actin caps in differentiating cells, we first examined the status of nuclear lamina protein lamin A/C. Indeed, our earlier work has shown that wild-type MEFs have an actin cap, while MEFs deficient in lamin A/C do not [13]. Here we found that lamin A/C was undetectable in undifferentiated hESCs (Fig. 5A left panels; the left corner of the colony in Fig. 5A-top left has started differentiating). As soon as TRA-1-81 was downregulated, lamin A/C was properly localized near the nuclear envelope (Fig. 5A, top left, corner of colony, and middle (1 day) and right panels (2 days)). In differentiation conditions, the hESCs that were still TRA-1-81-positive after 1 and 2 days in differentiation conditions continued to show no organized lamin A/C (Fig. 5A, middle and right panels). Together with the fact that *LMNA* knockout MEFs show no actin cap [13], these results suggest that the formation of an actin cap in hESCs follows the expression and proper organization of nuclear protein lamin A/C, which is repressed in undifferentiated hESCs.

We reasoned that the absence of organized lamin A/C in undifferentiated stem cells would result in the absence of the LINC complexes, which link the nuclear lamina to the cytoskeleton [6], at the nuclear envelope. Indeed, LINC complexes are disrupted in lamin A/C-deficient MEFs and in cells harboring disease-causing *LMNA* mutations [26]. We examined the status of LINC complex components Nesprin2 giant (Nuance) [27], [28], which has an actin-binding domain, Nesprin3, which binds F-actin through the large multi-domain protein plectin [29], [30], and Sun2, which links Nesprins to the nuclear lamina in the periplasmic space of the nuclear envelope [6], [31]. Immunofluorescence microscopy showed that Nesprin2 giant and Nesprin3 were undetectable in undifferentiated hESCs (Fig. 5, B and C). Sun2 was properly localized at the nuclear envelope before and after onset of differentiation, suggesting that Sun2 localization at the nuclear envelope does not require the actin cap or lamin A/C (Fig. 5D).

Within a day after initiation of differentiation, lamin A/C was organized at the nuclear envelope of TRA-1-81-negative hESCs (Fig. 5A, middle panels), and both Nesprin2 giant and Nesprin3 were properly localized in these cells (Fig. 5, B and C, middle panels). hESCs that were still TRA-1-81-positive in differentiation conditions continued to lack both organized lamin A/C and Nesprins at the nuclear envelope (Fig. 5 A–C). Moreover, all hESCs immuno-positive for lamin A/C were also immuno-positive for Nesprin2 giant and Nesprin3 and no hESCs immuno-negative for Nesprin2 giant or Nesprin3 were immuno-positive for lamin A/C (Fig. 5). Together with our previous finding that actin cap formation in somatic cells requires both lamin A/C and undisrupted LINC complexes [13], these results indicate that the perinuclear actin cap begins to form and becomes organized in differentiating hESCs when both lamin A/C and LINC complexes are expressed and properly organized at the nuclear envelope.

Since actin caps are also absent from hiPSCs, we determined whether lamin A/C and LINC complex components Nesprin2 giant and Sun2 were also absent from hiPSCs and present in parental HLFs, from which hiPSCs were derived. Immunofluorescence indicated that, similar to hESCs, hiPSCs showed no lamin A/C and no Nesprin2 giant at the nuclear envelope (Fig. 5, E and F, bottom rows). *Vice versa*, these molecules were properly localized at the nuclear envelope of parental HLFs (Fig. 5, E and F, top rows). Similar to hESCs, Sun2 was present and properly localized in hiPSCs (Fig. 5G). These results further support our model of formation of the actin cap in somatic and differentiating cells: actin cap formation in differentiating cells requires proper localization of LINC complexes and nuclear lamin A/C, while undifferentiated cells (hESCs and hiPSCs) show no actin cap because lamin A/C is absent, and accordingly, LINC complex components Nesprin2 giant and Nesprin3 that tether F-actin in the actin cap to the nuclear envelope, are absent in undifferentiated cells.

### Nuclear shaping by the perinuclear actin cap in hESCs and hiPSCs

**Figure 6:**
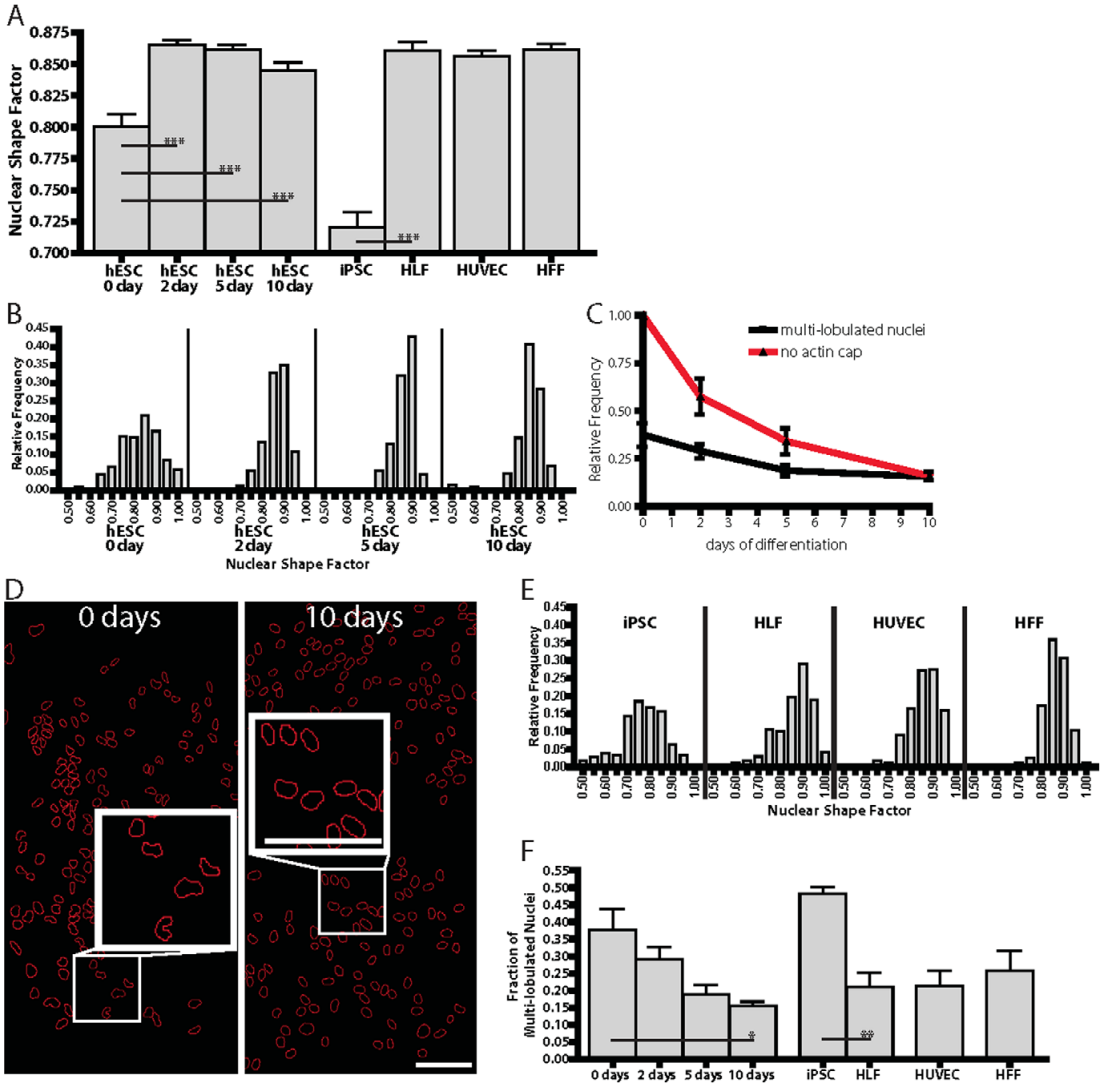
Nuclear shaping during hESCs undergoing differentiation. A. Ensemble-averaged nuclear shape factors of undifferentiated hESCs and cells undergoing differentiation, as well as iPSCs, parental HLFs, HUVECs, and HFFs. The shape factor is close to zero for a highly elongated nucleus and unity for a perfectly round nucleus. B. Distributions of nuclear shape factors in undifferentiated hESCs and cells undergoing differentiation. At least 200 cells were probed in triplicate. C. Fraction of multi-lobulated hESCs, with a nucleus featuring at least one lobe (black curve), and showing no actin cap (red curve) as a function of days following onset of differentiation. D. Typical shapes of nuclei in undifferentiated hESCs (left panel) and cells 10 days after onset of differentiation (right panel). Scale bar, 100 µm. E. Distributions of nuclear shape factors in iPSCs, their parental HLFs, HUVECs, and HFFs. F. Fractions of multi-lobulated nuclei in undifferentiated hESCs, hESCs undergoing differentiation, iPSCs, their parental HLFs, HUVECs, and HFFs. *: P<0.05; **: P<0.01.

Stem cells are characterized by pliable, ill-shaped nuclei [4]. Moreover, in MEFs, a critical function of the perinuclear actin cap is to control the shape of the interphase nucleus [13]. Therefore we asked whether the appearance of an actin cap shaped the nucleus in differentiating cells. Nuclear morphometric analysis was used to measure the mean and distribution of shape factors of nuclei in hESCs before and after induction of differentiation. The shape factor, defined as 4πA/P^2^ (A and P are the nuclear area and perimeter, respectively), approaches 1 for a rounded nucleus and approaches 0 for an elongated nucleus. Visual inspection of nuclei in undifferentiated hESCs, which have no actin caps, and differentiated hESCs, which do, revealed profound differences. Nuclei in undifferentiated hESCs were both multi-lobulated (i.e. with more than one lobe) and elongated; while nuclei in differentiating hESCs typically had a smooth morphology and a round shape (Fig. 6, A–E). Nuclear morphometry indicated that the progressive formation of perinuclear actin caps in differentiating hESCs coincided with a progressive enhancement of nuclear shape regularity, as measured by progressively higher nuclear shape factor (Fig. 6A) and progressively lower fraction of multi-lobulated nuclei (Fig. 6C). Moreover, while undifferentiated hESCs showed a wide distribution of nuclear shape factors, this distribution progressively narrowed overtime in differentiating conditions (Fig. 6B). Similar to cells at 10-day differentiation, HFFs and HUVECs exhibited high averaged nuclear shape factors (Fig. 6A), narrow distributions of nuclear shape factors (Fig. 6E), and fewer multi-lobulated nuclei (Fig. 6F). While the mean values of nuclear shape factor of iPSCs and hESCs were somewhat different, the distributions of nuclear shape factors were similar (Fig. 6, B and E), and the fractions of multi-lobulated nuclei (Fig. 6F) were similar as well. hESCs and iPSCs are both characterized by few DAPI bright spots (AT-rich, heterochromatic DNA) and a large nucleus to cytoplasmic ratio. The nuclear structure here, combined with the lack of lamin A/C and a perinuclear actin cap may have an effect on gene regulation and expression.

Next, we compared the shape of nuclei in iPSCs, which do not form a perinuclear actin cap, to the shape of nuclei in parental HLFs, which form a perinuclear actin caps. The trend of the average nuclear shape factor of hiPSCs from “undifferentiated” state to “differentiated” HLF's was similar to that of hESCs through the progression of differentiation (Fig. 6A). Similar to the nuclei of hESCs, nuclei of hiPSCs were multi-lobulated (Fig. 6F). As positive controls, the nuclear shape factors of parental HLFs, was found to be as high to the nuclear shape factors of HFFs and HUVECs (Fig. 6A). Moreover, while parental HLFs showed a narrow distribution of nuclear shape factors, the nuclear shape distribution became wide for iPSCs (Fig. 6E). Finally, parental HLFs showed few multi-lobulated nuclei (Fig. 6F). These highly consistent results between hiPSCs and hESCs suggest that the progressive formation of the actin cap mediates the progressive shaping of the nucleus – i.e. elimination of multiple lobes and nuclear rounding – in human pluripotent cells undergoing differentiation.

### Lamin A/C is required for the normal differentiation of mouse embryonic stem cells (mESC) and the formation of the perinuclear actin cap

To further investigate our proposed mechanism that lamin A/C, LINC proteins and, subsequently, the perinuclear actin cap are all required for proper differentiation, we investigated the differentiation of *Lmna^+/+^*, *Lmna^+/−^*, and *Lmna^−/−^* mouse embryonic stem cells [32]. Differentiation was induced by embryoid body (EB) formation and cells were collected at different time points for immunofluorescence.

**Figure 7:**
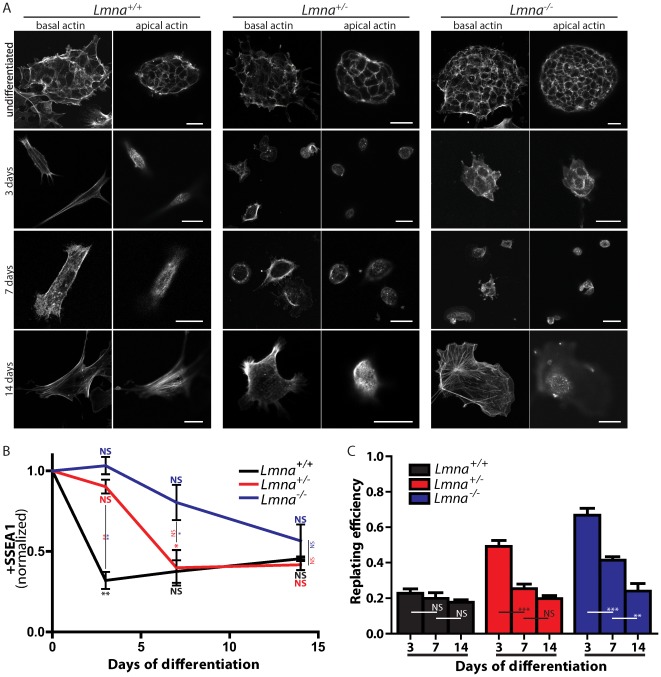
Lamin A/C is required for the proper differentiation of mouse embryonic stem cells (mESCs). A. Representative micrographs of basal (left columns) and apical (right columns) actin of *Lmna^+/+^*, *Lmna^+/−^*, and *Lmna^−/−^* mouse embryonic stem cells at day 0 (top row), at 3 days of differentiation (middle row), and after 14 days of differentiation (bottom row), illustrating the earlier appearance of the actin cap in wildtype cells (by 3 days) when compared to heterozygotes (∼7 days) and knockouts (>14 days). All scale bars: 20 µm. B. Flow cytometry analysis of normalized stage-specific embryonic antigen 1 (SSEA1) levels of *Lmna^+/+^*, *Lmna^+/−^*, and *Lmna^−/−^* mESCs through 14 days of differentiation. C. Replating efficiencies of *Lmna^+/+^*, *Lmna^+/−^*, and *Lmna^−/−^* mESCs after 3, 7, and 14 days of differentiation. *: P<0.05; **: P<0.01. ***: P<0.001; ns: non-significant difference.

First, we investigated the actin architecture of *Lmna^+/+^*, *Lmna^+/−^*, and *Lmna^−/−^* mESCs (Fig. 7A). At day 0, these cells grew in colonies and the organization of the actin filament network was similar, showing no hint of organized apical actin (Fig. 7A, top row). By day 3 of EB formation, *Lmna^+/+^* cells had taken on the familiar “stretched” actin architecture and showed the beginnings of the perinuclear actin cap (Fig. 7A, left column, second row). However, neither *Lmna^+/−^* nor *Lmna^−/−^* cells showed any organized perinuclear actin cap formation and mostly showed conventional cortical actin only (Fig 7A, second row, middle and right columns). At day 7, *Lmna^+/+^* cells looked similar to those at day 3. *Lmna^+/−^* cells started to spread more, and *Lmna^−/−^* were still small and contained mostly cortical actin. Neither *Lmna^+/−^* nor *Lmna^−/−^* cells showed any hint of an actin cap. By day 14, the majority of *Lmna^+/+^* cells showed a perinuclear actin cap, while fewer heterozygotes, and almost no knockouts showed a perinuclear actin cap (Fig. 7A, last row). These results show that, similar to human stem cells, mouse embryonic stem cells undergoing differentiation show a progressively more organized perinuclear actin cap and that its formation is regulated by lamin A/C.

Next, to investigate the progression of differentiation of the three cell types, we examined the levels of stage specific antigen 1 (SSEA1), a marker of pluripotency in mESCs. *Lmna^+/+^* cells were ∼30% positive at day 3 and remained at that level through day 14, while *Lmna^+/−^* and *Lmna^−/−^* mESCs took 7 and 14 days, respectively (Fig. 7B) to reach the same level of SSEA1 expression. As a final measure of the completeness of differentiation, we assessed the replating efficiency of each cell type. Results were consistent with the above data: wildtype mESCs reached their final level of replating efficiency (completeness of differentiation) (∼20%) by day three, while the heterozygotes (7 days) and the knockouts cells (14 days) took longer to reach the same level (Fig. 7C).

## Discussion

The results in this study suggest that the LINC complexes and the actin cap are intimately involved in stem cell differentiation and that their presence and proper localization at the nuclear envelope are likely required for normal development. While Nesprin 2 and Nesprin 3 appear to be expressed in undifferentiated mouse and human pluripotent cells, they do not localize to the nuclear envelope until lamin A/C is expressed, after the induction of differentiation. Proper localization of lamin A/C and LINC complex molecules at the nuclear envelope is followed by the formation of the perinuclear actin cap.

Our results indicate that the shape of the nucleus of pluripotent cells undergoing differentiation becomes progressively smoother, with fewer lobes, over a ten-day period of time (Fig. 6C). This timeline does not correlate with the rapid organization of nuclear lamin A/C and LINC complexes at the nuclear envelope, which occurs within one day of differentiation (Fig. 5, A–C). Instead, the nuclei of undifferentiated hESCs and iPSCs are misshapen because these cells do not feature a perinuclear actin cap. Clearly, the nuclear lamina (and in particular lamin A/C) provides the nucleus with some intrinsic stiffness [4], [33], [34], [35]. However, while the proper organizations of the nuclear lamina and LINC complexes at the nuclear envelope are required for actin cap formation, they are not sufficient to control the shape of the nucleus [13]. In differentiation conditions as in terminally differentiated somatic cells, the localization of lamin A/C and LINC complexes at the nuclear envelope mediate the formation of highly ordered stress fibers at the apical surface of the nucleus, which shape the nucleus.

We note that it is remarkable that the expression of four genes coding for transcription factors (*Oct4*, *Sox2*, *NANOG*, and *LIN28*) [36] in somatic cells have not only a dramatic effect on a precise and well defined subset of actin filament bundles, those forming the actin cap, which occur through the disruption of LINC complexes and lamin A/C. More work is needed to map the pathway connecting these genes to the lamin/LINC/Nesprin/actin-cap module. Through complex image processing of actin micrographs, Treiser *et al*. have recently shown that one can map the progress of cells through differentiation [37]. Here, we show that the most significant and qualitative change in actin organization during the course of differentiation is the perinuclear actin cap. Further work will need to be required to fully understand the different differentiation routes taken by cells lacking lamin A/C and which, if any, functions are dependent on nucleoskeletal connections.

The results in this paper add to the increasing list of structrual and functional differences between actin cap fibers and conventional stress fibers [5], [13]. Structural differences include: (*i*) the perinuclear actin cap is anchored to the nuclear envelope through LINC complexes as opposed to the plasma membrane in the case of conventional stress fibers; (*ii*) the perinuclear actin cap is composed of highly parallel, thick fibers as opposed to globally disorganized fibers at the basal cell surface; (*iii*) the perinuclear actin cap is made of fibers that are more contractile than conventional stress fibers [13]; (*iv*) the perinuclear actin cap is completely absent in undifferentiated pluripotent cells, in which conventional stress fibers are already formed. Functional differences include: (*v*) the perinuclear actin cap plays a critical role in shaping the interphase nucleus [13]; (*vi*) the perinuclear actin cap is absent in cells harvested from mouse models progeria and muscular distrophy, while the same cells show regular conventional stress fibers [13]; (*vii*) the perinuclear actin cap is involved in stem cell differentiation, not conventional actin fibers.

## Materials and Methods

### Human ESCs and iPSCs

Human ESC line H9 (passages 27 to 45; WiCell Research Institute, Madison, WI) and hiPSC line MP2 (passages 30 to 40; kindly provided by Dr. Linzhao Cheng [25]) were grown on an inactivated mouse embryonic feeder layer (Globalstem, Rockville, MD) in growth medium consisting of 80% ES-DMEM/F12 (Globalstem) supplemented with 20% knockout serum replacement and basic fibroblast growth factor (bFGF; both from Invitrogen, Carlsbad, CA) at concentrations of 4 ng/ml and 10 ng/ml for hESCs and hiPSCs, respectively.

### Mouse ESCs

*Lmna^+/+^, Lmna^+/−^, Lmna^−/−^* mouse embryonic stem cells (Gifts from Dr. Colin S. Stewart, ref. [30]) were grown on mitomycin-c treated primary mouse embryonic fibroblasts (Millipore, Billerica, MA) in growth medium consisting of: KO DMEM (Gibco, Carlsbad, CA) supplemented with 10% FBS (Gibco), 100 µm Non-Essential Amino Acids (Gibco), 0.1 mM β-Mercaptoethanol (Gibco), 1% Penicillin-Streptomycin (Gibco), 2 mM L-glutamine (Gibco), and with 1000 units/ml Human Recombinant Leukemia Inhibitory Factor (LIF) (Millipore).

### hESC differentiation

To induce differentiation, hESCs were digested with TrypLE (Invitrogen). Cells were separated into an individual cell suspension using a 40-µm mesh strainer. The individual hESCs were plated onto collagen-type-IV-coated plates (R&D Systems, Minneapolis, MN) in a concentration of 5×10^4^ cells/cm^2^. These cells were cultured in endothelial growth media (EGM; PromoCell, Heidelberg, Germany) with 50 ng/ml VEGF_165_ (Pierce, Rockford, IL) for ten days. Media were changed every second day.

### mESC differentiation

To induce differentiation, mESCs were digested with .25% Trypsin (Invitrogen) and then seeded in low-attachment dishes (Corning, NY) and supplemented with mESCs growth medium (described above) without the supplemental 1000 units/ml LIF.

### mESC replating efficiency

After 3, 7, and 14 days of differentiation, embryoid bodies were collected, trypsinized, counted, and replated on feeder cells in mESC medium. After one week, cells were fixed and stained with alkaline phosphatase (AP) according to manufacturer specifications (Millipore, Billerica, MA). Percentages reported are (colonies AP-stained/cells seeded)*100.

### Immunofluorescence microscopy

Actin filament and focal adhesion architecture were examined by immunofluorescence brightfield and confocal microscopy. Samples were fixed with 3.7% paraformaldehyde for 1 h, and stained for nuclear DNA, filamentous actin, tumor recognition antigen 1–81 (TRA-1-81), and nuclear envelope proteins lamin A/C, Nesprin2 giant, Nesprin3, and Sun2. For staining, cells were permeabilized with 0.1% Triton X-100 for 10 min. Goat serum, 10%, in phosphate-buffered saline was used to block nonspecific binding for 20 min. The primary antibodies used were: anti-TRA-1-81 antibody (Millipore MAB4381, Billerica, MA) at 1∶100; anti-lamin A/C (Abcam AB26300, Cambridge, MA) at 1∶500; anti-Sun2 (provided by Dr. Didier Hodzic, Washington University School of Medicine, at St. Louis) and anti-Nesprin 3 (provided by Dr. A. Sonnenberg, The Netherlands Cancer Institute, Amsterdam, The Netherlands) at 1∶2000 and 1∶1000, respectively; and anti-Nesprin 2 giant (provided by Drs. E. Gomes and G.G. Gundersen, Columbia University, New York) at 1∶500. Secondary treatments were done with Alexa-Fluor goat-anti-rabbit 488 or 568. Both primary and secondary antibody treatments were conducted for 1 h. To visualize actin filaments and nuclear DNA, Alexa-Fluor phalloidin 488 or 568 and 300 nM DAPI (Invitrogen, Carlsbad, CA) were used, respectively.

Fluorescent images were either collected using a Cascade 1 K CCD camera (Roper Scientific, Tucson, AZ) mounted on a Nikon TE2000E microscope with a 60× Plan Fluor objective (N.A. 1.4) or using a Zeiss 510 laser-scanning confocal microscope with a 63× Plan-Apochromat objective (N.A. 1.4). Three-dimensional images were analyzed and processed using a combination of Zeiss LSM Image Browser (Zeiss), Metaporph, and ImageJ (NIH). Special attention was paid to use small increments between focal sections (<0.3 µm) and to scan the same cell starting at slightly different heights as to not miss actin structures underneath the nucleus.

DAPI-stained nuclei were individually traced by hand and size, length of minor axis, length of major axis, and shape factor were measured using Metamorph software (Universal Imaging, Downingtown, PA). Mean values, standard error of measurement (SEM), and statistical analysis were calculated and plotted using Graphpad Prism (Graphpad Software, San Diego, CA). Two-tailed unpaired t tests were conducted to determine significance.

### Flow cytometry

Undifferentiated hESCs, day 5 and day 10 of differentiated hESCs were treated with EDTA for 5 min, counted, and separated into approximately 1×10^6^ cells per vial. They were then incubated separately in mouse anti-human TRA-1-60-FITC (BD Biosciences) or mouse IgM-FITC isotype control (BD Biosciences) for 1 h. Cells were rinsed twice with PBS, suspended in 0.1% bovine serum albumin and taken to the flow cytometry machine. User guide instructions were followed to complete the FACS analysis

### Statistics

Mean values, standard error of measurement (SEM), and statistical analysis were calculated using Graphpad Prism (Graphpad Software, San Diego, CA). Two-tailed unpaired *t* tests and ANOVA tests were conducted to determine significance, which was indicated using standard Michelin Guide scale (*** for *P*<0.001, ** for *P*<0.01, and * for *P*<0.05).

## Supporting Information

Figure S1 fraction of cells that are TRA-160 positive as a function of number of days after onset of differentiation of hESCs.
(TIFF)
