# Supplementary figures and images for: The Differential Formation of the LINC-Mediated Perinuclear Actin Cap in Pluripotent and Somatic Cells
Source: PLoS One. 2012 May 4;7(5):e36689. doi: 10.1371/journal.pone.0036689 (PMC3344930; doi:10.1371/journal.pone.0036689)

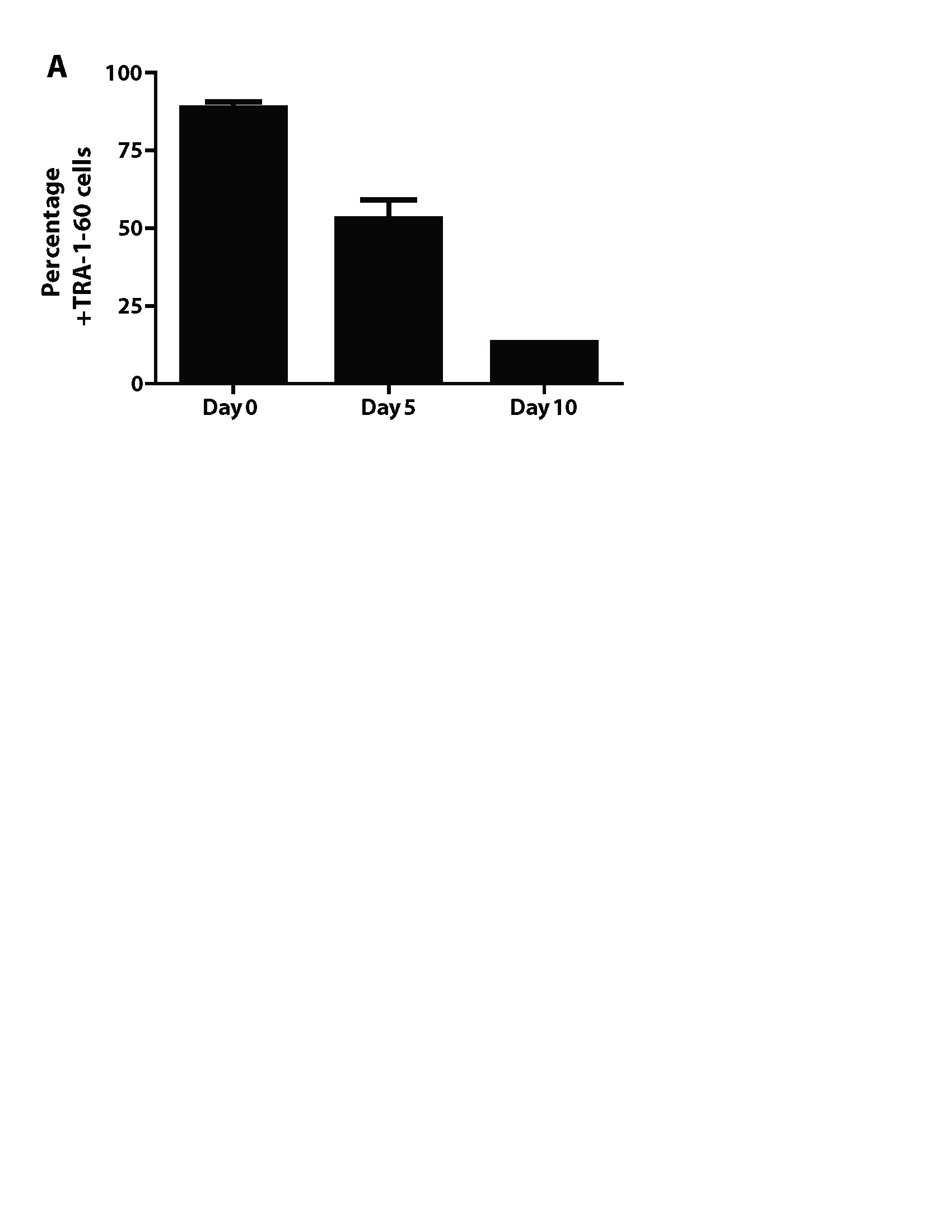

Supplement: Figure S1 — fraction of cells that are TRA-160 positive as a function of number of days after onset of differentiation of hESCs. (TIFF) [file pone.0036689.s001.tiff]
